# Conflito de interesses e influência da indústria nos ensaios clínicos randomizados na cirurgia da mão: uma revisão sistemática

**DOI:** 10.1055/s-0046-1824733

**Published:** 2026-07-28

**Authors:** Gabriel Henrique dos Santos Guimarães, Ramon Sampaio Souza Santos, Luís Renato Nakachima, Deivid Ramos dos Santos, João Carlos Belloti, Vinícius Ynoe de Moraes

**Affiliations:** 1 Departamento de Ortopedia e Traumatologia, Escola Paulista de Medicina, Universidade Federal de São Paulo, São Paulo, SP, Brasil; 2 Departamento de Ortopedia e Traumatologia, Programa de Residência em Ortopedia e Traumatologia, Universidade do Estado do Pará, Belém, PA, Brasil

**Keywords:** conflito de interesses, financiamento de capital, indústria, mão/cirurgia, ortopedia, revisão sistemática, capital financing, conflict of interest, hand/surgery, industry, orthopedics, systematic review

## Abstract

**Objetivo:**

Verificar a associação entre a presença de conflito de interesses e resultados pró-financiadores em ensaios clínicos randomizados (ECRs) de cirurgia da mão. Como objetivo secundário, avaliar a influência do financiamento da indústria sobre os dados bibliométricos desses ECRs.

**Métodos:**

Examinamos 178 ECRs publicados entre 2013 e 2023 em 5 periódicos de alto impacto com foco na mão, punho, cotovelo ou nervos periféricos, nas bases de dados MEDLINE, Embase e Scopus. Dos estudos incluídos, avaliamos a presença de conflito de interesses declarado e analisamos sua associação com o desfecho primário (favorável vs. não favorável). Avaliamos adicionalmente a associação com outras variáveis, incluindo conformidade com as diretrizes dos Padrões Consolidados de Relatórios de Ensaios (CONSORT, do inglês Consolidated Standards of Reporting Trials), número de autores e fator de impacto dos periódicos. O risco de viés dos estudos incluídos foi avaliado utilizando a ferramenta Risk of Bias 2 (RoB 2). Todas as avaliações foram realizadas por dois revisores independentes, seguindo protocolo previamente definido. Esta revisão sistemática não foi previamente registrada em plataformas públicas, como a International Prospective Register of Systematic Reviews (PROSPERO).

**Resultados:**

Não encontramos relação entre presença de conflito de interesses e resultados pró-financiadores (35,4% vs. 65,6%; *p* = 0,503). Estudos financiados pela indústria foram publicados em periódicos com fatores de impacto significativamente maiores do que estudos não financiados pela indústria (3,05 vs. 2,2) ( *p* < 0,001), com diferença significativa no número de autores financiados (1,84 vs. 0,07) ( *p* < 0,001).

**Conclusão:**

Não foi observada associação significativa entre o tipo de financiamento e a obtenção de resultados favoráveis em ensaios clínicos randomizados na cirurgia da mão. Estudos financiados foram mais frequentemente publicados em periódicos de maior impacto e apresentaram maior adesão ao *checklist* CONSORT, mas essas características não se traduziram em maior risco de viés.

## Abstract (en)

**Objective:**

To verify the association between the presence of conflict of interests and pro-funder results in randomized controlled trials (RCTs) in hand surgery. The secondary objective was to evaluate the influence of industry funding on the bibliometric characteristics of these RCTs.

**Methods:**

We examined 178 RCTs published between 2013 and 2023 in 5 high-impact journals focusing on the hand, wrist, elbow, or peripheral nerves in the MEDLINE, Embase, and Scopus databases. From the studies included, we assessed the presence of a declared conflict of interests and analyzed its association with the primary outcome (favorable vs. unfavorable). We additionally evaluated the association with other variables, including compliance with the Consolidated Standards of Reporting Trials (CONSORT) guidelines, the number of authors, and journal impact factors. The risk of bias in the included studies was assessed using the Risk of Bias 2 (RoB 2) tool. All assessments were performed by two independent reviewers, following a previously defined protocol. This systematic review was not previously recorded on public platforms such as International Prospective Register of Systematic Reviews (PROSPERO).

**Results:**

We found no relationship between the presence of conflict of interests and pro-funder results (35.4% vs. 65.6%. *p* = 0.503). Industry-funded studies have been published in journals with significantly higher impact factors than non-industry-funded studies (3.05 vs. 2.2) ( *p* < 0.001), with a significant difference in the number of funded authors (1.84 vs. 0.07) ( *p* < 0.001).

**Conclusion:**

There was no significant association between the type of funding and the achievement of favorable results in randomized clinical trials in hand surgery. Funded studies were more frequently published in higher impact journals and showed greater adherence to the CONSORT checklist, but these characteristics did not translate into a greater risk of bias.

## Introdução

A investigação clínica de qualidade requer, entre vários itens, aporte financeiro e o apoio da indústria em tornar reais seus produtos. Conflito de interesses (COI) é definido como “um conjunto de condições em que o julgamento profissional relativo a um interesse primário (como o bem-estar de um paciente ou a validade da pesquisa) tende a sofrer influência por um interesse secundário (por exemplo o ganho financeiro). Desse modo, o público-alvo dos artigos acadêmicos deve estar consciente do âmbito da influência da indústria. 1 2

O COI financeiro inclui, entre outros, subsídios/apoio à pesquisa, atuação como conselheiro, consultor ou defensor público, apoio financeiro indireto (por exemplo, reembolso de viagens, equipamentos), propriedade de ações, honorários pessoais, emprego direto, honorários por palestras, redação ou revisão do tema discutido no manuscrito, escritórios de palestrantes ou membros do conselho, royalties e patentes (planejadas, pendentes ou emitidas), independentemente do valor envolvido. O COI não financeiro podem incluir, entre outros, opiniões e relacionamentos pessoais, institucionais e profissionais que possam prejudicar a objetividade científica. 3

Uma das principais preocupações na investigação do COI é que a empresa que financia um ensaio pode ter um impacto nos resultados do ensaio. As diretrizes dos Padrões Consolidados de Relatórios de Ensaios (CONSORT, do inglês Consolidated Standards of Reporting Trials) exigem a divulgação do financiamento do estudo. O Comitê Internacional de Editores de Revistas Médicas (ICMJE, do inglês International Committee of Medical Journal Editors) também recomenda a divulgação de laços financeiros entre autores e patrocinadores. Embora a presença de COI ou divulgações de financiamento não impeça a consideração do artigo para publicação, a equipe editorial reserva-se o direito de recusar um artigo se considerar que o método científico tenha falhas ou que o financiador tenha comprometido a integridade do estudo. 3 4

Estudos anteriores 1 2 5 6 descobriram associação do patrocínio da indústria com maior probabilidade de reportar resultados e conclusões de eficácia favoráveis, bem como a taxas mais elevadas de discordância entre os resultados e as conclusões do estudo, em comparação com seus homólogos não patrocinados pela indústria, além de supressão de resultados negativos. 2 5 6 7

Vários estudos reportaram seus resultados acerca do tema nas especialidades médicas e não médicas (clínica médica, cirurgia geral, cirurgia plástica, ortopedia, fisioterapia). 1 3 8 9 10 11 12 Embora tenham explorado essa relação em outras especialidades, a pesquisa em cirurgia da mão carece de investigação suficiente sobre como o financiamento da indústria e o COI afetam os resultados e conclusões relatados.

Na declaração presumida de COI, justifica-se a hipótese de que existe relação entre a declaração explícita de COI e resultados positivos para os desfechos (primários, secundários, favorecimento do dispositivo). O objetivo primário do presente estudo foi verificar a associação entre a presença de COI com resultados pró-financiadores em ensaios clínicos randomizados (ECRs) de cirurgia da mão. O

## Métodos

A presente revisão sistemática foi elaborada com base nas diretrizes da declaração Preferred Reporting Items for Systematic reviews and Meta-Analyses (PRISMA). 13 Trata-se de um estudo secundário, conduzido de forma independente por pesquisadores vinculados a centros de referência em cirurgia da mão. Para garantir a imparcialidade da seleção e análise dos dados, não houve identificação institucional durante nenhuma etapa do processo metodológico.

### Estratégia de busca e seleção dos artigos

A pesquisa foi feita através da base de dados MEDLINE via Pubmed, Embase e SCOPUS, de acordo com protocolos semelhantes de estudos anteriores. 1 6 12 Utilizamos os descritores *surgery of the hand* OU *hand surgery* OU *surgery of the hands* OU *hand's surgery* OU *surgeries of the hand* . Foram incluídos apenas artigos publicados em inglês, português ou espanhol, por serem os idiomas mais prevalentes nas bases consultadas e de domínio dos revisores, assegurando a acurácia da extração e análise dos dados. As buscas foram realizadas entre os dias 1 e 31 de março de 2024. Os artigos foram selecionados por dois revisores de forma independente. Cinco importantes revistas cirúrgicas foram escolhidas com base no fator de impacto (FI), indexação internacional e reconhecimento acadêmico junto à comunidade da cirurgia da mão: *The Journal of Bone & Joint Surgery* (FI 5.3); *Journal of Hand Surgery* (FI 2.3); *The Journal of Hand Surgery* (European Volume) (FI 1.8); *HAND* (1.8); e *Revista Brasileira de Ortopedia* (FI 1.2).

Os títulos e resumos dos artigos identificados foram selecionados para excluir aqueles claramente irrelevantes para o foco da revisão. Os artigos selecionados na triagem inicial de título/resumo foram analisados.

### Critérios de inclusão/exclusão

Foram incluídos ECRs publicados entre 2013 e 2023, revisados por pares, com temática relacionada à cirurgia da mão, punho, cotovelo e nervos periféricos (doravante referidos coletivamente como “artigos sobre mão”). Foram excluídos editoriais, cartas ao editor, comentários, erratas e outros tipos de publicações não científicas. Os critérios adotados para a definição de um ECR incluíram: participação de seres humanos vivos no estudo; análise de uma intervenção relacionada aos cuidados de saúde; presença de um grupo comparativo; e alocação dos participantes por meio de randomização.

Nos casos em que o desfecho primário não estava explicitamente definido, o favorecimento foi determinado com base na análise das seções de resultados e conclusões, por dois revisores independentes. Em caso de discordância, a decisão final foi tomada por um terceiro revisor com experiência metodológica, mediante consenso.

### Desfechos específicos

Os artigos foram avaliados quanto à adesão ao CONSORT, COI declarado para os autores do artigo, número de autores (incluindo os financiados), tamanho da amostra e fonte de apoio ao estudo.

O financiamento de cada estudo foi categorizado como financiado pela indústria, não financiado pela indústria (outros financiamentos) ou sem financiamento. Um estudo foi considerado patrocinado pela indústria se pelo menos um autor estivesse listado como funcionário de uma indústria de saúde, ou se o apoio financeiro de uma empresa farmacêutica fosse reconhecido. Um estudo foi considerado não patrocinado pela indústria se fosse financiado por uma entidade não industrial (por exemplo, uma agência governamental ou instituição acadêmica). Os estudos que não receberam qualquer patrocínio foram considerados como sem financiamento. Os estudos que receberam financiamento da indústria ou de outras fontes não industriais foram categorizados, em ambos os casos, como estudos com algum tipo de financiamento.

Os resultados narrativos e as conclusões foram designados como *favoráveis* ou *desfavoráveis* . Na avaliação da seção de resultados, foi atribuído desfecho primário/secundário *favorável* aos ECRs que obtiveram resultados positivos com significância estatística. Desfecho *desfavorável* foi atribuído quando foram reportados resultados negativos. Na avaliação das seções de conclusão, o desfecho foi considerado *favorável* quando os autores declararam ou sugeriram favorabilidade à intervenção alvo, e *desfavorável* quando os autores declararam ou sugeriram favorabilidade em relação ao grupo de comparação ou controle. Em casos que houve conflito sobre o favorecimento da intervenção, um terceiro avaliador determinou a positividade.

## Análise do risco de viés

O risco de viés dos estudos incluídos foi avaliado com base na ferramenta RoB 2 (Risk of Bias 2.0), proposta pela Cochrane Collaboration, a qual contempla 5 domínios: (1) viés decorrente do processo de randomização; (2) viés devido a desvios da intervenção pretendida; (3) viés por dados ausentes; (4) viés na mensuração do desfecho; e (5) viés no relato seletivo dos resultados.

Cada domínio foi classificado como baixo risco de viés, alguma preocupação ou alto risco de viés, gerando um julgamento global para cada estudo. A avaliação foi realizada com base nas informações metodológicas e de resultados descritas nos artigos.

### Concordância interavaliadores

A categorização dos estudos quanto à favorabilidade dos desfechos, presença de COI, adesão ao *checklist* CONSORT e julgamento do risco de viés metodológico (RoB 2) foi realizada de forma independente por dois revisores com formação metodológica. Para todas essas variáveis de natureza subjetiva, a concordância entre os julgamentos foi avaliada por meio do coeficiente Kappa de Cohen, considerando as categorias atribuídas a cada estudo, nos quais valores abaixo de 0,40 indicam concordância fraca; entre 0,40 e 0,59, moderada; entre 0,60 e 0,79, substancial; e ≥ 0,80, quase perfeita.

Os resultados foram sintetizados em tabelas resumidas e utilizados em análises estratificadas para explorar possíveis associações entre risco de viés e tipo financiamento.

## Plano estatístico

O teste de análise de variância (ANOVA) foi usado para analisar a relação entre financiamento, autoria e variáveis quantitativas, incluindo número de autores, fator de impacto da revista e tamanho amostral. O teste do Qui-quadrado foi utilizado para analisar as relações entre financiamento, adesão ao CONSORT, declaração de COI, desfechos primários e secundários, conclusão qualitativa (favorável ou desfavorável à intervenção) e classificação global do risco de viés (RoB 2).

As análises estatísticas foram realizadas utilizando os softwares IBM SPSS Statistics for Windows (IBM Corp.), Versão 26.0 (2019), Minitab 21.2 (Minitab, LLC) (2022) e Microsoft Excel Office (Microsoft Corp.) 2010. Foi adotado nível de significância estatística de *p* < 0,05.

## Resultados

**Fig. 1:**
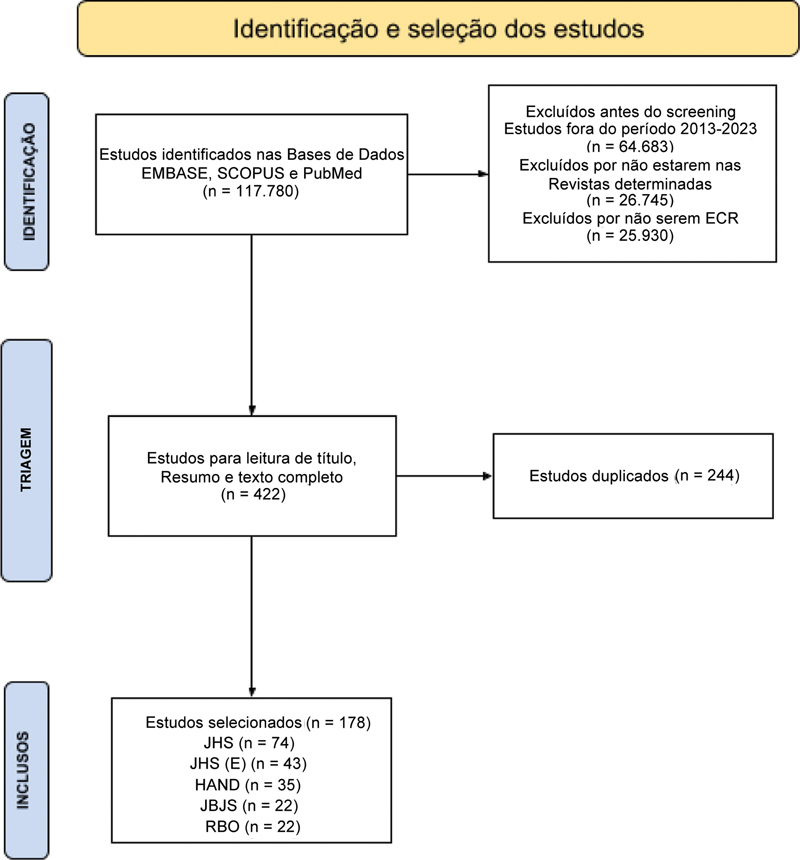
Fluxograma conforme protocolo Preferred Reporting Items for Systematic reviews and Meta-Analyses (PRISMA) para seleção dos estudos. **Abreviaturas** : JHS, *Journal of Hand Surgery* ; JHSE, *Journal of Hand Surgery* (European Volume); JBJS, The *Journal of Bone & Joint Surgery* ; RBO, *Revista Brasileira de Ortopedia* ; ECR, ensaio clínico randomizado.

Ao final da pesquisa, dos 117.780 estudos encontrados, foram incluídos 178 ECRs que correspondiam aos critérios de inclusão ( Fig. 1 ).

Não encontramos relação entre presença de COI e resultados pró-financiadores, sejam eles primários (35,4% vs. 65,6%) ou secundários (68,5% vs. 31,5%), favoráveis ou não ao dispositivo teste (68,6% vs. 31,4%) ( Tabelas 1 2 3 ).

**Tabela 1:** Relação do favorecimento do desfecho primário com a presença de financiamento e conflito de interesses

		Desfavorável		Favorável		Valor de *p*
		N	%	N	%	
**Todos**	**Sem declaração COI**	72	65,5%	43	63,2%	0,764
	**Com declaração COI**	38	34,5%	25	36,8%	
**Outro financiamento**	**Sem declaração COI**	8	27,6%	5	33,3%	0,692
	**Com declaração COI**	21	72,4%	10	66,7%	
**Financiado pela indústria**	**Sem declaração COI**	2	11,8%	0	0%	0,274
	**Com declaração COI**	15	88,2%	15	100%	
**Algum financiamento**	**Sem declaração COI**	10	21,7%	5	16,7%	0,587
	**Com declaração COI**	36	78,3%	25	83,3%	
**Sem financiamento**	**Sem declaração COI**	62	96,9%	38	100%	0,271
	**Com declaração COI**	2	3,1%	0	0%	

**Abreviatura:** COI, confito de interesses.

**Tabela 2:** Relação do favorecimento do desfecho secundário com a presença de financiamento e conflitos de interesse

		Desfavorável		Favorável		Valor de *p*
		N	%	N	%	
**Todos**	**Sem declaração COI**	85	68,5%	29	54,7%	0,078
	**Com declaração COI**	39	31,5%	24	45,3%	
**Outro financiamento**	**Sem declaração COI**	12	36,4%	1	9,1%	0,086
	**Com declaração COI**	21	63,6%	10	90,9%	
**Financiado pela indústria**	**Sem declaração COI**	1	5,9%	1	6,7%	0,514
	**Com declaração COI**	16	94,1%	14	93,3%	
**Algum financiamento**	**Sem declaração COI**	13	26,0%	2	7,7%	0,057
	**Com declaração COI**	37	74,0%	24	92,3%	
**Sem financiamento**	**Sem declaração COI**	72	97,3%	27	100%	0,388
	**Com declaração COI**	2	2,7%	0	0%	

**Abreviatura:** COI, conflito de interesses.

**Tabela 3:** Relação da conclusão favorável à intervenção com a presença de financiamento e conflitos de interesse

		Desfavorável		Favorável		Valor de *p*
		N	%	N	%	
**Todos**	**Sem declaração COI**	59	68,6%	56	60,9%	0,281
	**Com declaração COI**	27	31,4%	36	39,1%	
**Outro financiamento**	**Sem declaração COI**	7	30,4%	6	28,6%	0,892
	**Com declaração COI**	16	69,6%	15	71,4%	
**financiado pela Indústria**	**Sem declaração COI**	1	9,1%	1	4,8%	0,466
	**Com declaração COI**	10	90,9%	20	95,2%	
**Algum financiamento**	**Sem declaração COI**	8	23,5%	7	16,7%	0,455
	**Com declaração COI**	26	76,5%	35	83,3%	
**Sem financiamento**	**Sem declaração COI**	51	98,1%	49	98,0%	0,978

**Abreviatura:** COI, conflito de interesses.

Na avaliação das variáveis bibliométricas de base, demonstrou-se significância estatística no que diz respeito ao número de autores financiados (média de 1,34 para *outro financiamento* , 1,84 para *indústria* e 0,07 em *sem financiamento* ) e fator de impacto da revista (2,5 vs. 3,05 vs. 2,2) ( Tabela 4 ).

**Tabela 4:** Dados Bibliométricos dos estudos em relação ao tipo de financiamento dos estudos

Dados bibliométricos	Financiamento	N	Média	Mediana	Desvio padrão	IC 95%	Valor de *p*
**Num. autores**	Outro financiamento	44	4,93	5	1,35	4,53–5,33	0,241
	Indústria	32	5,53	6	2,08	4,81–6,25	
	Sem financiamento	102	5,00	5	1,68	4,67–5,33	
**Número de autores financiados**	Outro financiamento	44	1,34	0	2,10	0,72–1,96	< 0,001
	Indústria	32	1,84	1	2,26	1,06–2,62	
	Sem financiamento	102	0,07	0	0,38	0–0,14	
**Fator de impacto**	Outro financiamento	44	2,50	2,3	1,15	2,16–2,84	0,001
	Indústria	32	3,05	2,3	1,45	2,55–3,55	
	Sem financiamento	102	2,20	1,8	0,89	2,03–2,37	
**Tamanho da amostra**	Outro financiamento	44	88,43	64,5	76,67	65,78–111,08	0,627
	Indústria	32	83,31	58,5	63,71	61,24–105,38	
	Sem financiamento	102	78,30	65	47,67	69,05–87,55	

**Abreviatura:** COI, conflito de interesses.

Comparando os três grupos, a análise das variáveis qualitativas “ *adesão ao CONSORT* e “ *declaração de COI* demonstrou significância estatística ( Tabela 5 ). Estudos financiados pela indústria mostraram uma taxa de adesão ao CONSORT de 56,3% em comparação a outros estudos financiados (50%) e estudos não financiados (28,4%) ( *p* = 0,004). Já o índice positivo de *declaração de COI* foi de 93,8%, 70,5% e 2,0% nos respectivos grupos ( *p* < 0,001).

**Tabela 5:** Característica dos estudos em relação ao tipo de financiamento

Característica dos estudos		Outro financiamento		Indústria		Sem financiamento		Total		Valor de *p*
		N	%	N	%	N	%	N	%	
**Adesão ao CONSORT**	Não	22	50,0%	14	43,8%	73	71,6%	109	61,2%	0,004
	Sim	22	50,0%	18	56,3%	29	28,4%	69	38,8%	
**Declaração de COI**	Não	13	29,5%	2	6,3%	100	98,0%	115	64,6%	< 0,001
	Sim	31	70,5%	30	93,8%	2	2,0%	63	35,4%	
**Desfecho primário**	Desfavorável	29	65,9%	17	53,1%	64	62,7%	110	61,8%	0,503
	Favorável	15	34,1%	15	46,9%	38	37,3%	68	38,2%	
**Desfecho secundário**	Desfavorável	33	75,0%	17	53,1%	75	73,5%	125	70,2%	0,064
	Favorável	11	25,0%	15	46,9%	27	26,5%	53	29,8%	
**Resultado favorável ao dispositivo**	Desfavorável	23	52,3%	11	34,4%	52	51,0%	86	48,3%	0,217
	Favorável	21	47,7%	21	65,6%	50	49,0%	92	51,7%	

**Abreviaturas:** COI, conflito de interesses; CONSORT, Consolidated Standards of Reporting Trials.

A aplicação da ferramenta RoB 2 permitiu avaliar o risco de viés dos estudos incluídos ( Tabela 6 ). Considerando o grande número de estudos incluídos, optou-se por apresentar os resultados de forma agregada. Os dados detalhados da avaliação por estudo e por domínio estão disponíveis conforme descrito na seção de disponibilidade dos dados. O coeficiente de concordância interavaliadores (Kappa de Cohen) foi de 0,79, sugerindo concordância substancial. A maioria dos estudos foi classificada como de baixo risco de viés (90/178; 50,6%), seguida por alguma preocupação (73/178; 41,0%) e alto risco (15/178; 8,4%). Quando estratificados por tipo de financiamento, observou-se distribuição semelhante entre os grupos: por exemplo, estudos com financiamento da indústria apresentaram 50% de baixo risco, 40,6% de alguma preocupação e 9,4% de alto risco. A análise estatística não identificou associação significativa entre financiamento e risco de viés ( *p* = 0,537 para *sem financiamento* ; *p* = 0,996 para *algum financiamento* ).

**Tabela 6:** Análise global do risco de viés (RoB 2) estratificado por categoria de financiamento nos estudos incluídos (n = 178)

Tipo de financiamento	Análise do risco de viés (Rob 2)				
	Baixo risco	Alguma preocupação	Alto risco	Total	Valor de *p*
Sem financiamento	51	43	8	102	0,537 a
Financiamento da indústria	16	13	3	32	
Outro financiamento	24	16	4	44	
**Algum financiamento**	**39**	**30**	**7**	**76**	0,996 a
**Todos**	**90**	**73**	**15**	**178**	**0,79** b

**Notas:**^a^ teste Qui quadrado de Pearson. ^b^ Coeficiente de concordância interavaliadores (Kappa de Cohen) = 0,79.

## Discussão

Em nossa amostra, os estudos financiados pela indústria foram, em média, publicados em periódicos com maior fator de impacto (3,05; IC 95% 2,55–3,55), quando comparado aos estudos sem financiamento (média de 2,2; IC 95% 2,03–2,37), com diferença estatística significativa ( *p* < 0,001). Não foi observada diferença estatisticamente significante entre estudos com *Outros Financiamentos* (média 2,5; IC 95% 2,16–2,84) em relação aos demais grupos.

Diversos autores já alertaram sobre a influência potencial da indústria sobre o processo científico, não apenas nos resultados, mas também no interesse editorial e na maior propensão à publicação de achados favoráveis aos dispositivos estudados. 6 14 15 16 17

No entanto, quanto à avaliação da positividade dos desfechos primários entre os grupos sem e com financiamento ( *p* = 0,503) no nosso estudo, não foi possível descartar a hipótese de que ambos os grupos teriam proporções de positividade semelhantes. Nesse contexto, o desfecho primário foi positivo em 46,9% dos estudos financiados pela indústria e 37,3% dos estudos sem financiamento, sem haver diferença estatisticamente significante. O mesmo ocorreu para o desfecho secundário ( *p* = 0,064) e Resultado Favorável ao dispositivo ( *p* = 0,217).

Esses achados contrastam com a literatura prévia, como demonstrado por Bhandari et al., 6 que identificaram maior proporção de resultados estatisticamente significantes pró-financiadores em ECRs cirúrgicos financiados.

Resultados similares também foram descritos em áreas como neurocirurgia, 18 dermatologia 19 e cirurgia da coluna. 14 Nesse último estudo, Munsch et al. 14 por exemplo, relataram maior proporção de desfechos positivos em estudos financiados pela indústria, inclusive em relação a subgrupos financiados por agências públicas, além de identificarem viés de publicação seletiva de estudos com resultados negativos.

Já em cirurgia plástica, observou-se equilíbrio entre estudos financiados e não financiados na área reconstrutiva, mas maior proporção de financiamento industrial na cirurgia estética. 12 Esse padrão se aproxima do perfil observado em nosso estudo, voltado à cirurgia da mão, uma área majoritariamente reconstrutiva.

Na ortopedia, Nesello et al. 20 também encontraram maior proporção de resultados positivos em estudos financiados pela indústria que utilizaram plasma rico em plaquetas, especialmente entre estudos de menor nível de evidência. Em contraste, em nosso estudo, não houve diferença significativa entre os grupos quanto ao tamanho amostral, embora o número de autores tenha sido maior nos estudos com financiamento ( *p* < 0,05).

No que diz respeito à qualidade do reporte, identificamos maior adesão ao checklist CONSORT entre os estudos financiados pela indústria (56,3%), seguidos por outros financiamentos (50%) e sem financiamento (28,4%)—diferença estatisticamente significativa ( *p* = 0,004). O CONSORT reforça a transparência e reprodutibilidade dos estudos e, embora não seja obrigatório, sua adoção é vista como indicativo de rigor metodológico. O checklist do CONSORT apresenta 25 itens que auxiliam na transparência, reprodutibilidade e interpretação adequada dos resultados do estudo. 21 22 23 24

No entanto, nosso estudo também apresenta limitações relevantes. O viés de seleção das revistas, todas com elevado fator de impacto, pode ter favorecido a inclusão de ECRs com maior rigor metodológico, reduzindo a heterogeneidade da amostra. Além disso, ao limitar a seleção ao período pós-2013, pode ter havido maior prevalência de estudos submetidos a exigências mais estritas de transparência editorial.

Outro ponto crítico diz respeito à confiabilidade das declarações de COI. Trabalhos como os de Hannon et al. 25 e Tian et al. 26 identificaram discrepâncias significativas entre declarações autorais e registros de bancos de dados públicos, como o Open Payments. Esse tipo de subdeclaração pode mascarar a real extensão do financiamento e impactar a categorização de estudos.

Finalmente, ao avaliar o risco de viés metodológico (RoB 2), identificamos que a maioria dos estudos foi classificada como de baixo risco, com distribuição relativamente homogênea entre os grupos de financiamento. A análise estatística não indicou associação significativa entre o tipo de financiamento e o julgamento global do risco de viés ( *p* ≥ 0.05). Ainda assim, a presença de estudos com “alguma preocupação” em todas as categorias sinaliza que a atenção à qualidade metodológica deve ser constante, independentemente da fonte de financiamento.

Vale destacar, entretanto, que o RoB 2 pode não capturar completamente os vieses estruturais associados ao financiamento, como seleção de comparadores, definição de desfechos ou reporte seletivo. Como apontado por Lundh et al. 27 e reforçado por Devji et al., 5 estudos financiados pela indústria podem apresentar conclusões mais favoráveis ao patrocinador mesmo quando classificados como de baixo risco de viés, exigindo cautela na interpretação desses resultados.

Dessa forma, embora o financiamento não deva ser usado isoladamente como critério de julgamento da validade de um estudo, ele deve ser considerado no contexto do seu reporte, transparência metodológica e risco potencial de influenciar interpretações. Nosso trabalho contribui ao demonstrar que estudos financiados, quando bem desenhados e publicados em revistas com alto rigor editorial, não necessariamente apresentam maior risco de viés, mas continuam a exigir análise crítica cuidadosa.

### Limitações do estudo

O risco de viés de seleção de periódicos é relevante: a amostra foi composta exclusivamente por revistas com alto fator de impacto, o que pode ter excluído ECRs relevantes publicados em periódicos de menor visibilidade, potencialmente enviesando a análise em favor de estudos com maior rigor metodológico e maior frequência de financiamento declarado.

Além disso, a categorização do financiamento e dos COIs foi baseada unicamente nas declarações dos autores, que podem estar sujeitas a omissões ou imprecisões—como já demonstrado por estudos que compararam essas declarações com registros públicos de pagamentos da indústria.

Por fim, a ausência de registro prévio do protocolo em plataformas públicas, como o International Prospective Register of Systematic Reviews (PROSPERO), constitui uma limitação do estudo.

## Conclusão

Este estudo não identificou evidências robustas de que o tipo de financiamento—seja pela indústria, por fontes independentes ou sem financiamento declarado—esteja associado a uma maior proporção de resultados favoráveis à intervenção em ensaios clínicos randomizados na área da cirurgia da mão. Observou-se que os estudos financiados foram, em média, publicados em periódicos com maior fator de impacto e apresentaram maior adesão ao checklist CONSORT, o que pode refletir maior rigor metodológico ou exigências editoriais mais estritas.

Apesar desses achados, recomenda-se cautela na interpretação, uma vez que a análise não foi ajustada para potenciais fatores de confusão e ferramentas como o RoB 2 podem não capturar plenamente vieses estruturais relacionados ao financiamento, definição de desfechos e reporte seletivo. Além disso, a inclusão de periódicos de alto fator de impacto pode ter limitado a variabilidade metodológica dos estudos avaliados.

Em síntese, embora o financiamento não tenha se associado diretamente a maior risco metodológico ou à tendência de resultados favoráveis, a influência potencial de patrocinadores no desenho, condução e reporte dos estudos permanece uma preocupação válida e deve continuar sendo objeto de escrutínio crítico nas futuras revisões sistemáticas e práticas editoriais.

## References

[1] Ross P R Wood S M Chung K C Industry Funding and Self-Declared Conflict of Interest in Hand Surgery Publications J Hand Surg Am 2020 45 06 479 487 10.1016/j.jhsa.2020.02.017 32245714

[2] Bridoux V Moutel G Schwarz L Michot F Herve C Tuech J J Disclosure of funding sources and conflicts of interest in phase III surgical trials: survey of ten general surgery journals World J Surg 2014 38 10 2487 2493 10.1007/s00268-014-2580-5 24824646

[3] El Moheb M Karam B S Assi L Armache M Khamis A M Akl E A The Policies for the Disclosure of Funding and Conflict of Interest in Surgery Journals: A Cross-Sectional Survey World J Surg 2021 45 01 97 108 10.1007/s00268-020-05771-0 32914281

[4] Itani K M Reda D J Clinical Trials Design in Operative and Non-Operative Invasive Procedures Cham, Switzerland Springer Nature 2017

[5] Devji T Busse J W Cochrane in CORR ^®^ : Industry Sponsorship and Research Outcome Clin Orthop Relat Res 2017 475 09 2159 2164 10.1007/s11999-017-5421-7 28634896 PMC5539042

[6] Bhandari M Busse J W Jackowski D Association between industry funding and statistically significant pro-industry findings in medical and surgical randomized trials CMAJ 2004 170 04 477 480 14970094 PMC332713

[7] Heigle B Shepard S Anderson J M Weaver M Hartwell M Vassar M The influence of industry sponsorship and conflict of interest on results and conclusions of systematic reviews regarding treatment of knee osteoarthritis Osteoarthr Cartil Open 2021 3 01 100142 10.1016/j.ocarto.2021.100142 36475067 PMC9718174

[8] Fuentes J Armijo-Olivo S Costa BRd Does Type of Sponsorship of Randomized Controlled Trials Influence Treatment Effect Size Estimates in Rehabilitation: A Meta-Epidemiological Study Am J Phys Med Rehabil 2020 99 10 909 916 10.1097/PHM.0000000000001444 32960528

[9] Clifford T J Barrowman N J Moher D Funding source, trial outcome and reporting quality: are they related? Results of a pilot study BMC Health Serv Res 2002 2 01 18 10.1186/1472-6963-2-18 12213183 PMC126226

[10] Printz J O Lee J J Knesek M Urquhart A G Conflict of interest in the assessment of hyaluronic acid injections for osteoarthritis of the knee: an updated systematic review J Arthroplasty 2013 28(8, Suppl)30 330 10.1016/j.arth.2013.05.034 23890521

[11] Probst P Grummich K Ulrich A Büchler M W Knebel P Diener M K Association of industry sponsorship and positive outcome in randomised controlled trials in general and abdominal surgery: protocol for a systematic review and empirical study Syst Rev 2014 3 138 10.1186/2046-4053-3-138 25431307 PMC4280764

[12] Momeni A Becker A Bannasch H Antes G Blümle A Stark G B Association between research sponsorship and study outcome in plastic surgery literature Ann Plast Surg 2009 63 06 661 664 10.1097/SAP.0b013e3181951917 19887933

[13] Tricco A C Lillie E Zarin W PRISMA Extension for Scoping Reviews (PRISMA-ScR): Checklist and Explanation Ann Intern Med 2018 169 07 467 473 10.7326/M18-0850 30178033

[14] Munsch M A Chen S R Dalton J Tisherman R Shaw J D Lee J Y Association Between Industry Sponsorship of Spine-Related Clinical Trials, Publication Status, and Research Outcomes Global Spine J 2024 14 07 2039 2044 10.1177/21925682231166379 37129370 PMC11418736

[15] Bekelman J E Li Y Gross C P Scope and impact of financial conflicts of interest in biomedical research: a systematic review JAMA 2003 289 04 454 465 10.1001/jama.289.4.454 12533125

[16] Kjaergard L L Als-Nielsen B Association between competing interests and authors' conclusions: epidemiological study of randomised clinical trials published in the BMJ BMJ 2002 325 7358 249 10.1136/bmj.325.7358.249 12153921 PMC117638

[17] Als-Nielsen B Chen W Gluud C Kjaergard L L Association of funding and conclusions in randomized drug trials: a reflection of treatment effect or adverse events? JAMA 2003 290 07 921 928 10.1001/jama.290.7.921 12928469

[18] Khan N R Saad H Oravec C S A Review of Industry Funding in Randomized Controlled Trials Published in the Neurosurgical Literature-The Elephant in the Room Neurosurgery 2018 83 05 890 897 10.1093/neuros/nyx624 29462484

[19] Perlis C S Harwood M Perlis R H Extent and impact of industry sponsorship conflicts of interest in dermatology research J Am Acad Dermatol 2005 52 06 967 971 10.1016/j.jaad.2005.01.020 15928613

[20] Nesello P FT Baroni A C Selistre LdS Level of Evidence and Industry Sponsorship Associated with Favorable Outcomes in Publications on Platelet-Rich-Plasma Therapy in Musculoskeletal Disorders Rev Bras Ortop 2020 55 03 263 268 10.1055/s-0039-1700834 PMC7316539 32616969

[21] Consolidated Standards of Reporting Trials Group Moher D Hopewell S Schulz K F CONSORT 2010 Explanation and Elaboration: Updated guidelines for reporting parallel group randomised trials J Clin Epidemiol 2010 63 08 e1 e37 10.1016/j.jclinepi.2010.03.004 20346624

[22] Junkin J C Vraa D Young J L Rhon D I Assessing the transparency in reporting of clinical trials investigating manual therapy interventions for low back pain: A methodological review J Eval Clin Pract 2024 30 08 1594 1602 10.1111/jep.14078 38973108

[23] Cuschieri S The CONSORT statement Saudi J Anaesth 2019 13 01 S27 S30 10.4103/sja.SJA_559_18 30930716 PMC6398298

[24] Teixeira R KC Pimentel A LJC Vasconcelos MEdSL Editorial policies of Brazilian journals about guidelines. Rev Assoc Med Bras (1992) Rev Assoc Med Bras 2021 67 03 345 346 10.1590/1806-9282.20200813 34468593

[25] Hannon C P Chalmers P N Carpiniello M F Cvetanovich G L Cole B J Bach B R Jr Inconsistencies Between Physician-Reported Disclosures at the AAOS Annual Meeting and Industry-Reported Financial Disclosures in the Open Payments Database J Bone Joint Surg Am 2016 98 20 e90 10.2106/JBJS.15.01119 27869631

[26] Tian T Shah A Y Darling J Assessment of self-reported financial conflicts of interest in vascular surgery studies J Vasc Surg 2021 74 06 2047 2053 10.1016/j.jvs.2021.05.040 34171423

[27] Lundh A Lexchin J Mintzes B Schroll J B Bero L Industry sponsorship and research outcome Cochrane Database Syst Rev 2017 2 02 MR000033 10.1002/14651858.MR000033.pub3 28207928 PMC8132492

